# Cardiovascular Care Among Cancer Survivors in the United States

**DOI:** 10.1093/jncics/pky049

**Published:** 2018-12-07

**Authors:** Kevin A Pearlstein, Ramsankar Basak, Ronald C Chen

**Affiliations:** 1 Department of Radiation Oncology; 2 Lineberger Comprehensive Cancer Center; 3 Sheps Center for Health Services Research, University of North Carolina at Chapel Hill, Chapel Hill, NC

## Abstract

**Background:**

Cardiovascular disease (CVD) is a leading cause of mortality among cancer survivors, but whether survivors receive routine cardiovascular monitoring and preventive care has not been well studied. This study uses a population-based dataset to examine this question.

**Methods:**

Data from the National Health Interview Survey were used to identify 13 266 cancer survivors who completed surveys from 2011 to 2015. Prevalence of CVD and associated risk factors, patterns of doctor visitation, and receipt of CVD preventive care were examined. We used multivariable logistic regression analysis to examine factors associated with the receipt of preventive care for survivors with and without CVD risk factors.

**Results:**

CVD risk factors were prevalent in older cancer survivors 65 years and older (56.9% with hyperlipidemia, 66.8% with hypertension) and younger survivors younger than 50 years (35.4% obese, 30.3% current smokers). Rates of blood pressure, cholesterol, and glucose monitoring were high, but rates of lifestyle modification were lower (54.8% moderate exercise, 47.1% smoking cessation attempts among smokers). Although 71.5% of survivors at 2 years or less from diagnosis saw both general and specialist doctors, only 51.6% of survivors at 5 or more years saw both, and 43.5% saw only a general doctor. On multivariable analysis, receipt of CVD preventive care was strongly associated with general doctor visitation for those with and without CVD risk factors.

**Conclusions:**

CVD and associated risk factors are prevalent among both older and younger cancer survivors across the United States. This study identifies areas for improvement related to lifestyle modification in survivors, and also highlights the importance of care transition to the primary care provider for long-term survivors.

With recent advances in cancer treatments, an increasing number of cancer patients are becoming long-term survivors. Approximately two-thirds of newly diagnosed cancer patients are now estimated to survive at least 5 years, and it is projected that by 2024 there will be nearly 19 million cancer survivors in the United States (1). National organizations have recognized that cancer survivors have unique healthcare needs (2); specifically, preventive care is described by the Institute of Medicine as an important component of healthcare for cancer survivors in their publication “From Cancer Patient to Cancer Survivor: Lost in Transition” (3).

Cardiovascular disease (CVD) is a leading cause of mortality in both the general population and among cancer survivors in the United States (4–9). National guidelines for the general population emphasize the importance of routine preventive care for CVD (10,11). Guidelines specific to cancer survivors are beginning to emerge (12), recognizing that this population may be at an even higher risk for developing CVD than the general population due to CVD that can be caused by certain types of systemic therapy and radiation therapy (12–17). Therefore, cardiovascular preventive care is equally important, and perhaps more so, among cancer survivors.

However, the receipt of cardiovascular preventive care has not been well studied among cancer survivors. We used a US population-based data source to examine the prevalence of CVD and the receipt of preventive care.

## Materials and Methods

### Source of Data

We used data from the National Health Interview Survey (NHIS), an annual cross-sectional survey designed by the National Center for Health Statistics, to provide data that reflects the overall health status and healthcare of the noninstitutionalized US population. A new sample of US households is interviewed each year regarding participants’ health status, sociodemographics, and healthcare utilization (18). These data are commonly used to represent healthcare patterns in the United States (19–21).

### Analytic Cohort

We examined the NHIS database to identify individuals who reported a history of cancer (excluding nonmelanomatous skin cancer). We identified 13 266 individuals available in NHIS survey years 2011–2015. Consistent with the definition provided by the Institute of Medicine (3) and used in previous NHIS studies (19,20), we defined the survivorship period as starting immediately upon cancer diagnosis.

### Statistical Analysis

Descriptive statistics were used to examine the prevalence of CVD and associated risk factors, incorporating NHIS sample weights to reflect population-based estimates, as was done in prior studies (19,20). We examined if patients saw a general doctor (defined as a doctor in general practice, family medicine, or internal medicine) and/or a specialist doctor (all other doctors) and the number of doctor visits in the past year. Further, based on the data available in NHIS, we examined specific CVD preventive care items, including rates of blood pressure, cholesterol, and fasting glucose checks. We also examined rates of lifestyle interventions that have been shown to reduce cardiovascular mortality in the general population (22), including exercise in the past month and, among those who reported tobacco use, recent attempts at smoking cessation. Multivariable logistic regression models were used to examine the factors associated with the receipt of blood pressure checks, cholesterol checks, and fasting glucose checks among cancer survivors with and without cardiovascular risk factors. Analysis was performed using SAS v. 9.4 (Cary, NC). A two-sided *P* value with a significance level of .05 was used.

## Results

Of the 13 266 cancer survivors included in this analysis, 42.9% were younger than 65 years (Table 1). A total 83.1% survivors were diagnosed more than 2 years prior to the NHIS survey and 55.9% were more than 5 years from their cancer diagnosis. The major cancers represented in this study were breast cancer (22.6%), gynecologic cancer (15.5%), and prostate cancer (14.2%). Cardiovascular risk factors were prevalent in this population, particularly among older individuals, where 66.8% reported hypertension and 56.9% reported hyperlipidemia (Table 2). Further, 39.8% of individuals older than 65 years reported a diagnosis of CVD. Risk factors were also prevalent among younger survivors, particularly active smoking (30.3%) and obesity (35.4%).

**Table 1.** Demographic characteristics of cancer survivors (N = 13 266)

Characteristic	No. (%)
Age, y	
<40	893 (6.7)
40–49	1028 (7.8)
50–64	3771 (28.4)
65–69	1884 (14.2)
70–84	4451 (33.6)
≥85	1239 (9.3)
Race	
Non-white	2210 (16.7)
White	11 056 (83.3)
Sex	
Female	8031 (60.5)
Male	5235 (39.5)
Education	
Non-high school graduate	2088 (15.8)
High school graduate only	3712 (28.1)
Some college	3851 (29.2)
College graduate	3553 (26.9)
Region	
Midwest	3066 (23.1)
Northeast	2264 (17.1)
South	4669 (35.2)
West	3267 (24.6)
Marital status	
Married	5831 (44.0)
Not married	7412 (56.0)
Individual income	
<$35 000	2091 (44.3)
$35 000-$75 000	1283 (27.2)
>$75 000	683 (14.5)
Unknown	664 (14.1)
Health insurance	
Yes	12 244 (97.4)
No	326 (2.6)
Year of NHIS Survey	
2011	12 244 (97.4)
2012	2433 (18.3)
2013	2597 (19.6)
2014	2623 (19.8)
2015	2861 (21.6)
Type of cancer*	
Breast	3003 (22.6)
Colorectal	1057 (8.0)
Prostate	1887 (14.2)
Gynecologic	2053 (15.5)
Other	5528 (41.7)
Time from cancer diagnosis, y	
≤2	2154 (16.9)
3-5	3454 (27.2)
≥6	7107 (55.9)

* Sums over 100 because of multiple cancer incidences.

**Table 2.** Cardiovascular disease and risk factors among cancer survivors stratified by age*

Medical condition	Total	<50 y	50–64 y	>65 y
CVD risk factors				
Hypertension	53.2%	27.6%	48.7%	66.8%
Cholesterol	48.4%	22.4%	44.4%	56.9%
Diabetes	17.7%	8.0%	17.2%	22.1%
Current smoker	14.0%	30.3%	20.1%	7.8%
Obesity	30.4%	35.4%	35.3%	26.8%
CVD				
Any CVD	29.8%	14.5%	23.7%	39.8%
Stroke	6.4%	3.6%	5.4%	8.9%
MI	8.7%	2.7%	6.7%	12.2%
Angina	5.5%	1.8%	4.6%	7.0%
Heart condition, unspecified	17.4%	9.8%	14.1%	22.1%

* Analysis incorporated NHIS sample weights to reflect population-based estimates of the prevalence of each studied condition. CVD = cardiovascular disease; MI = myocardial infarction.

Most cancer survivors reported general doctor visitation in the past year (86.3%), whereas fewer reported specialist doctor visitation (59.4%) (Table 3). Although most survivors (71.5%) 2 years or less from diagnosis saw both general and specialist doctors in the past year, this decreased over time and longer-term survivors were more likely to see a general doctor only (Table 4). A total 51.6% of survivors of more than 5 years saw both general and specialist doctors, and 43.5% saw only a general doctor. Nearly all survivors reported blood pressure checks (95.9%) and cholesterol checks (86.0%). Individuals with 0–1 cardiovascular risk factors had lower rates of preventive care screening than those with known CVD or more than 1 risk factor: blood pressure checks (94.1% vs 98.0%), cholesterol checks (79.9% vs 93.2%), and fasting glucose checks (53.0% vs 69.4%). Rates of lifestyle modification were more modest, with 54.8% reporting moderate exercise within the past month, and only 47.1% of current smokers reporting a recent attempt at smoking cessation.

**Table 3.** Healthcare utilization and preventive care among cancer survivors*

Doctor visitation	
General doctor visit in past year	86.3%
Specialist doctor visit in past year	59.4%
Number of doctor visits in past year	
0	5.0%
1–3	29.2%
≥4	65.8%
Preventive care	
Blood pressure check in past year	95.9%
Cholesterol check in past year	86.0%
Fasting glucose check in past year	60.5%
Moderate exercise in past month	54.8%
Tried to quit smoking in past year†	47.1%

* Analysis incorporated NHIS sample weights to reflect population-based estimates.

† Among individuals who currently smoke (*N* = 2021).

**Table 4.** Healthcare utilization by time from cancer diagnosis

Doctor visitation in past 12 months	All	≤2 years from diagnosis	3–5 years from diagnosis	>5 years from diagnosis
General doctor visit only	35.1%	21.1%	30.8%	43.5%
Specialist doctor visit only	5.9%	7.4%	6.6%	4.9%
Both general and specialist doctor visit	59.0%	71.5%	62.6%	51.6%

On multivariable analysis among cancer survivors with CVD or risk factors, receipt of cardiovascular monitoring (blood pressure, cholesterol, glucose) was most strongly associated with general doctor visitation (Table 5). Findings were similar among cancer survivors without a history of cardiovascular risk factors (Table 6).

**Table 5.** Multivariable analysis of factors associated with blood pressure check, cholesterol check, and fasting glucose check in the past year among cancer survivors with cardiovascular disease or risk factors

Characteristic	Blood pressure check, OR (95% CI)	Cholesterol check, OR (95% CI)	Fasting glucose check, OR (95% CI)
General doctor visit			
No	REF	REF	REF
Yes	12.90 (8.85 to 18.79)*	4.41 (3.44 to 5.66)*	2.66 (2.19 to 3.23)*
Specialist doctor visit			
No	REF	REF	REF
Yes	2.43 (1.61 to 3.65)*	1.63 (1.34 to 1.98)*	1.33 (1.19 to 1.49)*
Age at survey (5 year increments)	1.05 (0.97 to 1.14)	1.15 (1.10 to 1.20)*	1.01 (0.98 to 1.04)
Race			
White	REF	REF	REF
Non-white	0.94 (0.53 to 1.65)	1.13 (0.84 to 1.50)	1.19 (1.01 to 1.39)*
Sex			
Female	REF	REF	REF
Male	0.84 (0.55 to 1.29)	1.30 (1.05 to 1.62)*	1.21 (1.07 to 1.38)*
Education			
College graduate	REF	REF	REF
< High school	0.79 (0.46 to 1.36)	0.88 (0.65 to 1.20)	0.85 (0.71 to 1.01)
High school	1.25 (0.78 to 2.02)	0.95 (0.72 to 1.26)	0.88 (0.75 to 1.03)
Some college	0.88 (0.53 to 1.47)	0.92 (0.69 to 1.23)	1.08 (0.92 to 1.26)
Region			
West	REF	REF	REF
Northeast	1.21 (0.66 to 2.22)	1.36 (0.95 to 1.97)	1.17 (0.95 to 1.44)
Midwest	1.34 (0.71 to 2.53)	1.06 (0.79 to 1.42)	1.15 (0.97 to 1.35)
South	1.19 (0.72 to 1.96)	1.23 (0.94 to 1.60)	0.99 (0.85 to 1.15)
Married			
No	REF	REF	REF
Yes	1.19 (0.79 to 1.77)	1.48 (1.20 to 1.84)*	1.15 (1.02 to 1.28)*
Health insurance			
No	REF	REF	REF
Yes	2.68 (1.26 to 5.71)*	2.19 (1.36 to 3.54)*	1.24 (0.84 to 1.83)

* *P* < .05 compared to reference category (REF). OR = odds ratio; CI = confidence interval.

**Table 6.** Multivariable analysis of factors associated with blood pressure check, cholesterol check, and fasting glucose check in past year in cancer survivors without cardiovascular disease or risk factors

Characteristic	Blood pressure Check, OR (95% CI)	Cholesterol check, OR (95% CI)	Fasting glucose check, OR (95% CI)
General doctor visit			
No	REF	REF	REF
Yes	15.08 (8.11 to 28.04)*	4.29 (3.01 to 6.12)*	3.03 (2.07 to 4.44)*
Specialist doctor visit			
No	REF	REF	REF
Yes	6.08 (3.04 to 12.14)*	1.49 (1.05 to 2.12)*	1.13 (0.85 to 1.49)
Age at survey (5 year increments)	0.95 (0.86 to 1.05)	1.16 (1.09 to 1.24)*	1.10 (1.05 to 1.16)*
Race			
White	REF	REF	REF
Non-white	0.84 (0.37 to 1.92)	1.66 (1.01 to 2.74)*	1.15 (0.76 to 1.72)
Sex			
Female	REF	REF	REF
Male	0.60 (0.33 to 1.10)	0.75 (0.53 to 1.06)	0.83 (0.63 to 1.09)
Education			
College graduate	REF	REF	REF
< High school	0.39 (0.15 to 1.04)	0.65 (0.36 to 1.16)	0.81 (0.47 to 1.39)
High school	0.33 (0.15 to 0.72)*	0.62 (0.39 to 0.99)*	0.60 (0.41 to 0.87)*
Some college	0.50 (0.22 to 1.11)	0.67 (0.44 to 1.02)	0.80 (0.57 to 1.11)
Region			
West	REF	REF	REF
Northeast	0.94 (0.37 to 2.43)	0.80 (0.46 to 1.37)	0.78 (0.51 to 1.21)
Midwest	0.85 (0.38 to 1.90)	0.60 (0.36 to 0.98)*	0.82 (0.56 to 1.19)
South	0.81 (0.39 to 1.67)	0.70 (0.47 to 1.06)	0.66 (0.45 to 0.97)*
Married			
No	REF	REF	REF
Yes	1.52 (0.87 to 2.66)	1.34 (0.92 to 1.97)	1.35 (1.03 to 1.76)*
Health insurance			
No	REF	REF	REF
Yes	2.07 (0.78 to 5.48)	1.62 (0.71 to 3.67)	1.84 (0.94 to 3.59)

* *P* < .05 compared to reference category (REF). OR = odds ratio; CI = confidence interval.

## Discussion

The importance of preventive care among cancer survivors has been emphasized by numerous groups, including the Institute of Medicine and the American Society of Clinical Oncology (2,3). Indeed, this study shows that CVD and risk factors are highly prevalent in cancer survivors across the United States in both younger and older survivor age groups. General doctor visitation was frequent and was strongly associated with the receipt of cardiovascular preventive monitoring, including blood pressure, cholesterol, and fasting glucose checks. Further, general doctor visitation remained stable in longer-term cancer survivors, while specialist doctor visitation decreased. The rate of exercise and smoking cessation attempts were relatively low.

CVD has been identified as a leading cause of mortality among survivors of breast cancer (4–6), prostate cancer (8), and other cancers (7). In addition to a baseline risk for CVD as experienced by the general, noncancer population, some cancer patients may have an even greater risk due to certain oncologic treatments such as anthracycline chemotherapy (23), androgen deprivation therapy (24), and thoracic radiotherapy (14). Preventive care for CVD has been shown to reduce mortality in the general population and is incorporated in national guidelines. For example, routine screening for hypertension has been designated as a Grade A recommendation by the US Preventive Services Task Force (10), and screening and treatment of high cholesterol as well as discussion of lifestyle modifications for overweight individuals with cardiovascular risk factors have been designated as Grade B recommendations (11,25). Among patients who have diagnosed hypertension or hyperlipidemia, medical interventions are effective and have been shown to reduce mortality, highlighting the importance of screening for these cardiovascular risk factors (26,27). Tobacco cessation has been associated with decreased mortality by up to 35% in certain populations (22). Exercise has also been found to reduce mortality in the general population by 20–30% (22,28). Taken together, these data highlight the importance of the preventive care items examined in this study.

Our finding that general doctor visitation was more common than specialist doctor visitation, particularly among long-term cancer survivors, and was strongly associated with preventive care warrants further discussion. These findings suggest an initial “shared care” of cancer survivors between the oncology specialist and primary care providers in the early years of survivorship, with gradual transition to primary care only over time. However, we were not able to distinguish oncologists from other specialists in the NHIS dataset. During this transition period, close communication and coordination among care providers is important to outline individual roles in the care of cancer survivors (29). As our results show, cancer survivors who do not see a primary care provider are much less likely to receive preventive care related to cardiovascular issues. This exemplifies a group of survivors that the Institution of Medicine described as “lost in transition” (3).

Our findings complement those from prior studies that used the Surveillance, Epidemiology, and End Results Medicare database, but the current study is unique in focusing on issues specific to cardiovascular preventive care. In prior studies using claims data, contact with primary care physicians was associated with receipt of general preventive services including influenza vaccination, bone density screening, and cancer screening (30–33). However, many items analyzed in the current study (blood pressure check, moderate exercise, attempt at smoking cessation) are not available in claims data. Together, the findings from prior studies and ours highlight the central role of primary care providers in the overall health of cancer survivors, and the importance of their involvement in the long-term care of cancer survivors.

Strengths of this study include the use a population-based dataset designed to reflect the health status and healthcare of the US population. Thus, the results of this study can be used to inform policy makers about the current state of survivorship care in the United States. Moreover, NHIS includes data for individuals both older and younger than 65 years; the latter is not included in other commonly used data such as Surveillance, Epidemiology, and End Results Medicare. Additionally, NHIS is unique in providing many preventive care items specific to CVD that are not available in many other population-based datasets. A limitation of NHIS is that it is composed of patient-reported data and is subject to nonresponse bias as well as potential misclassification of medical conditions and receipt of medical services. Despite this, data from NHIS are specifically recommended as a data source for monitoring the healthcare of the US population in the framework set forth by Healthy People 2020 (34) and have been used to evaluate issues such as cancer screening (35), smoking cessation (36), and other high-risk health behaviors (19). Additionally, although certain cancer treatments can increase the risk of CVD, this information is not available and therefore could not be incorporated in our analysis. The cross-sectional nature of NHIS does not allow for the evaluation of preventive care for preexisting cardiovascular issues separate from new cardiovascular issues after cancer treatment. Lastly, although we conclude that there is room for improvement for cancer survivors, a direct comparison was not made with noncancer individuals. who may have better or worse care than our study population.

In summary, CVD and its risk factors are highly prevalent among cancer survivors. Cardiovascular risk factors are not limited to the elderly and are present in many younger survivors. Our finding of a strong association between primary care visitation and improved cardiovascular preventive care highlights the importance of the transition of care during cancer survivorship back to the primary care provider. We also found opportunities to improve lifestyle interventions including exercise and smoking cessation in cancer survivors.

## Notes

Affiliations of authors: Department of Radiation Oncology (KAP, RB, RCC) and Lineberger Comprehensive Cancer Center (RCC) and Sheps Center for Health Services Research (RCC), University of North Carolina at Chapel Hill, Chapel Hill, NC.

## Funding

None of the authors report a conflict of interest. There was no external funding.
